# Global Survey of Alternative Splicing in Rice by Direct RNA Sequencing During Reproductive Development: Landscape and Genetic Regulation

**DOI:** 10.1186/s12284-021-00516-6

**Published:** 2021-08-12

**Authors:** Haoxuan Li, Aixuan Li, Wei Shen, Nenghui Ye, Guanqun Wang, Jianhua Zhang

**Affiliations:** 1 Department of Biology, Hong Kong Baptist University, Kowloon, Hong Kong; 2 School of Life Sciences and State Key Laboratory of Agrobiotechnology, The Chinese University of Hong Kong, Shatin, Hong Kong; 3 CAS Key Laboratory of RNA Biology, Institute of Biophysics, Chinese Academy of Sciences, Beijing, China; 4 Southern Regional Collaborative Innovation Center for Grain and Oil Crops in China, College of Agriculture, Hunan Agricultural University, Changsha, 410128 China

**Keywords:** *Oryza sativa*, Direct RNA sequencing, Alternative splicing, Small RNA targets, Transcription factors, Reproductive development

## Abstract

Alternative splicing is a widespread phenomenon, which generates multiple isoforms of the gene product. Reproductive development is the key process for crop production. Although numerous forms of alternative splicing have been identified in model plants, large-scale study of alternative splicing dynamics during reproductive development in rice has not been conducted. Here, we investigated alternative splicing of reproductive development of young panicles (YP), unfertilized florets (UF) and fertilized florets (F) in rice using direct RNA sequencing, small RNA sequencing, and degradome sequencing. We identified a total of 35,317 alternative splicing (AS) events, among which 67.2% splicing events were identified as novel alternative splicing events. Intron retention (IR) was the most abundant alternative splicing subtype. Splicing factors that differentially expressed and alternatively spliced could result in global alternative splicing. Global analysis of miRNAs-targets prediction revealed that alternative spliced transcripts affected miRNAs’ targets during development. Degradome sequencing detected only 6.8% of the differentially alternative splicing transcripts, suggesting a productive transcripts generation during development. In addition, alternative splicing isoforms of *Co-like,* a transcription factor, interacted with *Casein kinase 1-like protein HD1* (*CKI*) examined in luciferase assay, which could modulate normal male-floral organs development and flowering time. These results reveal that alternative splicing is intensely associated with developmental stages, and a high complexity of gene regulation.

**Supplementary Information:**

The online version contains supplementary material available at 10.1186/s12284-021-00516-6.

## Background

Alternative transcription initiation, splicing, polyadenylation and translation initiation are the four manners affecting gene expression levels, which determine protein diversity in eukaryotic cells interdependently (de Klerk and AC‘t Hoen 2015). Splicing is an enigmatic process in eukaryotic organisms, which spliced the precursor mRNA (pre-mRNA) in manners of intron excision and exon ligation, thus contributes to coding potential and functionality of multi-exonic genes in both animals and plants (Gelfman et al. 2012; James et al. 2012; Chang et al. 2014; Feng et al. 2015; Li et al. 2020). Components of the spliceosome include small nuclear ribonucleoproteins (snRNPs) and numerous Ser/Arg-rich (SR) proteins (Wahl et al. 2009; Will and Lührmann 2011). The genome of rice encodes 22 SR proteins which are essential in constitutive and alternative splicing of rice pre-mRNA (Isshiki et al. 2006). Alternative splicing (AS) sites determined by splice site sequences in exons and introns, which called splicing enhancers, are binded by different splicing factors, and subsequently caused the splicing events (Wang et al. 2008). Splicing factors with different expression levels or activities regulate AS through different splice sites, which produce more than one spliced mRNA from one gene (James et al. 2012). Commonly, five alternative splicing types are recognized in plants, namely intron retention (IR), exon skipping (ES), mutually exclusive exon (MEE), alternative 5′ splice sites (A5SS), and alternative 3′ splice sites (A3SS) (Barbazuk et al. 2008). Analysis of AS events are widely conducted in various plant species with the advent of sequencing approaches, for example, the next-generation and full-length sequencing (direct RNA sequencing (dsRNA-seq)). It has been reported that greater than 60% or 80% of intron-containing genes undergo AS in Arabidopsis (Filichkin et al. 2010; Zhu et al. 2017), Glycine max (soybean) (Aghamirzaie et al. 2013) and Zea mays (maize) (Thatcher et al. 2014, 2016). However, some other plants display less AS events in intron-containing genes, for example, ~ 53% or less in rice (Zhang et al. 2010; Dong et al. 2018; Chen et al. 2019) and ~ 24% in wheat (Liu et al. 2018b).

Alternative splicing has been demonstrated to play essential roles in a variety of different plant processes, including tissue identity and developmental stages (Staiger and Brown 2013; Thatcher et al. 2016). Evidence also shows that the AS pattern changes along with developmental stages (Iida et al. 2004). It’s proposed that tissue-specific AS results in alteration of subcellular localization, from cytoplasm to endoplasmic reticulum (Kriechbaumer et al. 2012). For example, gene of *viviparous1* (*Vp1*) regulates seed development through simultaneously activating embryo maturation and repressing germination, while it shows weak embryo dormancy and is susceptible to preharvest sprouting caused by the mis-splicing of *Vp-1* homologous in hexaploid wheat (*Triticum aestivum*) (McKibbin et al. 2002). Mutation of the *Waxy* gene of rice (*Oryza sativa*) encoding a granule bound starch synthase reduces splicing efficiency and thus results in lower levels of amylose content grains (Cai et al. 1998; Isshiki et al. 1998; Larkin and Park 1999). Moreover, AS also plays roles in modulating crucial developmental process, such as the flowering time and circadian clock (Lopato et al. 1999; Harmer et al. 2000; Ali et al. 2007; Hong et al. 2010; Streitner et al. 2010). Interaction of AS with other posttranscriptional processes in plants also lead to a variety of different isoforms. Recently, AS patterns have been verified to alter a transcript’s sensitivity, for example, the maize (Zea mays) *SPX* family produce miR827-sensitive or insensitive isoforms in different tissues (Thatcher et al. 2016). In addition, transcription of *FLOWERING LOCUS C*(*FLC*) was modulated by long non coding RNA (lncRNA) isoforms (Marquardt et al. 2014).

Rice yield is influenced by both genetic and epigenetic factors, including vegetative phase, and floret development (Ikeda et al. 2004). Sex cell development before fertilization, involving the pollen development before flowering (Berger and Twell 2011; Twell 2011) and embryo sac development before fertilization (Yadegari and Drews 2004; Dresselhaus 2006), and the developmental process of flowering and fertilization, in which pollen is transferred to the stigma where pollen tube germinates and enters the ovary and embryo sac, and releases a pair of sperm cells into the embryo sac (Berger and Twell 2011). After double fertilization, the egg cell develops into embryo and the central cell develops into endosperm. Therefore, the florets development process is complex and significant. Rice floret development has been intensively investigated for decades, whereas the regulating mechanism on this fundamental biological process is still far from well-illustrated. Investigations on the young panicle development, sex cell development before fertilization and post-fertilization in terms of AS are necessary to gain deeper insight into this complex process. Although genome-wide changes have been extensively investigated, the AS isoforms based on the direct RNA sequencing (dRNA-seq) during the rice floret development and the correlation between AS isoforms and miRNAs, lncRNAs are seldom reported. Figuring out the AS patterns of rice floret development from the young panicles to fertilized spikelets, and the significance of AS in modulating posttranscriptional regulation are crucial for rice production. Therefore, we mainly deploy direct dRNA-seq and degradome sequencing (degradome-seq), to illustrate the alternative splicing landscape and genetic regulation during rice reproductive development.

## Results

### Overview of Rice Transcripts Detected by dRNA-seq

Alternative splicing is a widespread phenomenon, which is essential for post-transcriptional regulation mediating the mRNA stability and protein diversity of eukaryotic genomes. In this study, we utilized the dRNA-seq technology to study the alternative splicing events in rice using young panicles (YP), unfertilized florets (UF) and fertilized florets (F). The dRNA-seq generated 2.01 million, 2.07 million and 1.80 million reads with a read N50 (the minimum contig length required to cover 50% of the assembled genome sequence) of 1080, 1095 and 1210, respectively, in the YP, UF and F. The mean length of the reads ranged from 706 to 818 bp, while the max length reached from 7063 to 8030 bp. After error correction, 1,761,906, 1,836,813 and 1,606,743 reads were mapped to the Rice MSU7.0 genomes, while the mapping rate was 87.62%, 88.60% and 89.40%, respectively. Then we analyzed the quality of the sequencing reads, which showed consistently high-quality scores over the length of reads (Additional file 1: Fig. S1A, B). The full-length transcript numbers ranged from 1,718,488 to 2,189,176, which accounted over 73.9% among the total clean read numbers (Additional file 2: Table S1). In total, 56,718 genes were annotated in the genome, and 1347 genes were annotated as new genes (Additional file 2: Table S2). The dRNA-seq generated 51,742 transcripts at least in one sample, among which 10,067 were new isoforms mapped to the known genes, and 1633 were considered as novel isoforms, which mapped to the new genes (Additional file 2: Table S2). We also calculated the mapping rate of all the novel genes and transcripts to rice and other plant species (Additional file 1: Fig. S2). Results showed that ~ 94.63% new genes were mapped to rice genome, and few new genes were mapped to other species (Additional file 1: Fig. S2A). The above novel genes mapped to rice genome were significantly enriched in various GO terms (Additional file 1: Fig. S2B). In total, 85.76% novel transcripts were mapped to the rice genome which significantly enriched in various GO terms (Additional file 1: Fig. S2C, D). However, few of the novel transcripts were mapped to other species (Additional file 1: Fig. S2C). Thus, the novel genes and transcripts distributed among various pathways during floret development.

### Identification of Full-Length Alternative Splicing Events by dRNA-seq

**Fig. 1:**
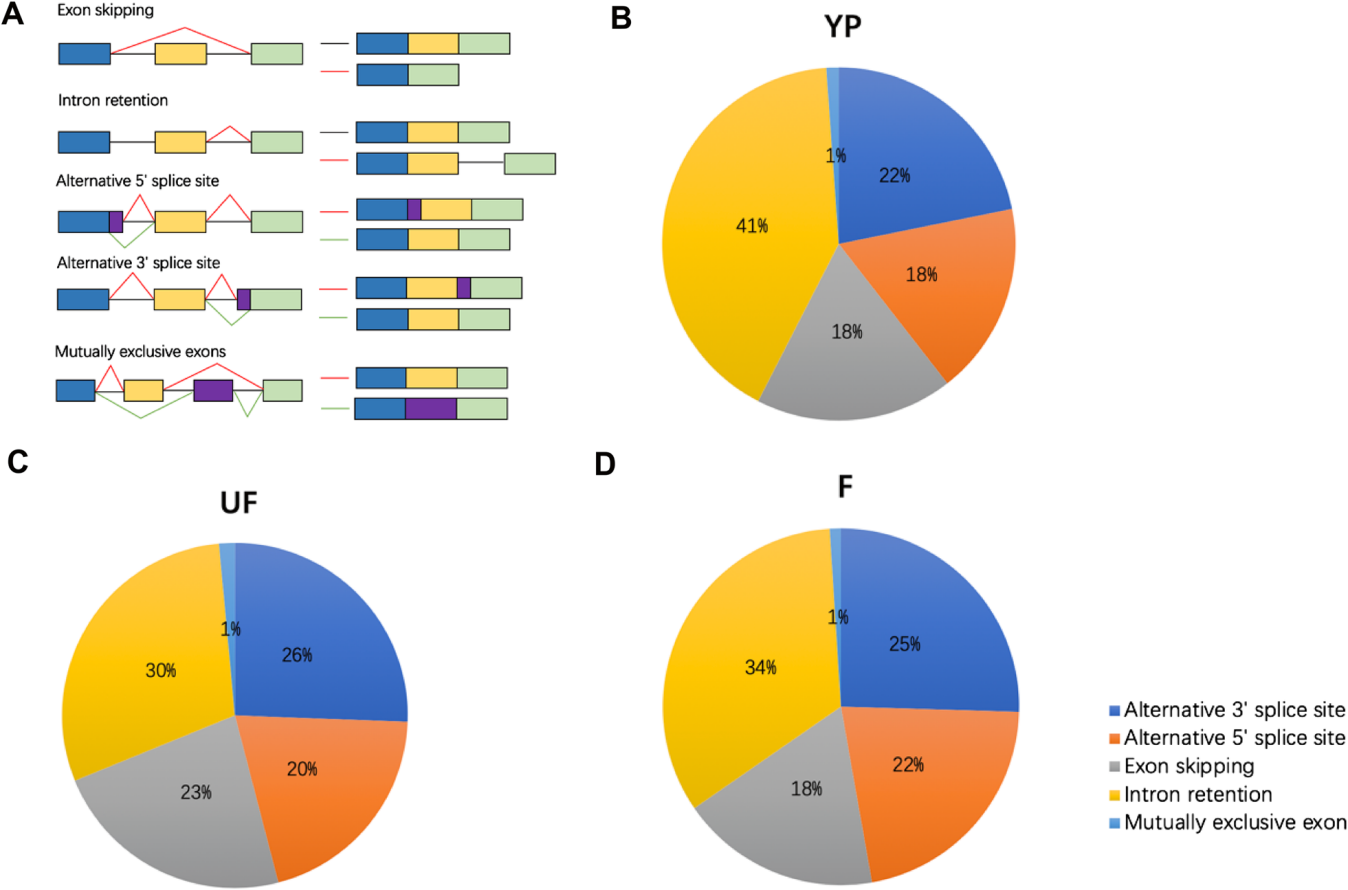
Alternative splicing landscapes of rice young panicles (YP), unfertilized florets (UF), and fertilized florets (F) based in the Nanopore sequencing data. **a** Alternative splicing (AS) types, namely Alternative 3′ splice site (A3SS), Alternative 5′ splice site (A5SS), Exon skipping (ES), Intron retention (IR), and Mutually exclusive exons (MEE). **b**–**d** Frequency of each AS types in sample of YP, UF and F, respectively

**Fig. 2:**
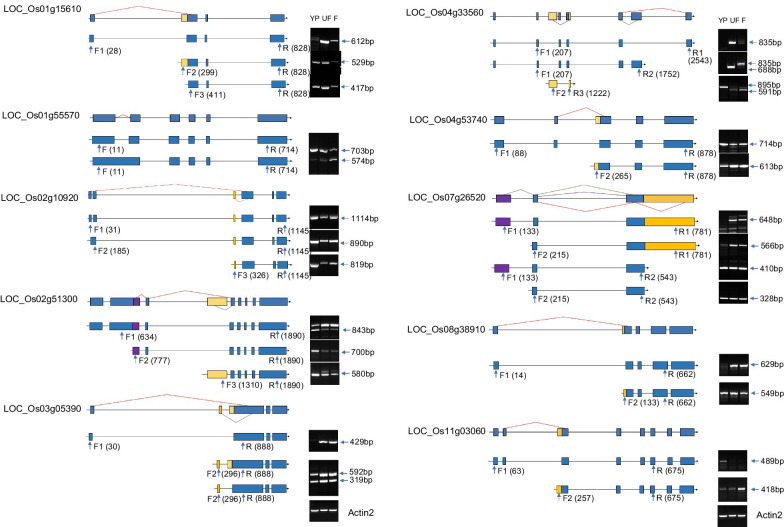
Validation of different alternative splicing (AS) events by RT-PCR. Fourteen AS isoforms were selected to check each isoform in RT-PCR and eleven AS were detectable at the mRNA level either with changed transcript levels or new isoforms. The primer of each hand used for variation were arrowed and the primer position numbered from transcription start sites ATG were indicated. The product size of each alternative isoform was showed. The cycles of RT-PCR were showed in Additional file 2: Table S3. The dRNA-seq data of the above DAS events were showed in Additional file 1: Figure S3. *Actin2* was used as the inner control

Alternative splicing events occurred in the three samples were defined as five major types of alternative splicing events, including Mutually exclusive exons (MEE), Intron retention (IR), Exon skipping (ES), Alternative 5′ splice site (A5SS) and Alternative 3′ splice site (A3SS) (Fig. 1a). We calculated all the alternative splicing events in each of the sample using astalavista (Foissac and Sammeth 2007) and the alternative splicing events were examined in the current annotation gene model. In total, we detected 35,317 alternative splicing events, in which 11,599 (~ 32.8%) splicing events were derived from the annotated genes, and 23,718 (67.2%) splicing events were identified as novel alternative splicing events originated from both annotated and unannotated genes. To figure out the distribution of alternative splicing types in different samples, we plotted the pie charts to present the percentages of each types. In the YP sample, IR was the most abundant alternative splicing events (41%), and A3SS was the second most abundant alternative splicing types (22%), then A5SS and ES comprised 18% (Fig. 1b). In contrast, alternative splicing types of IR (30%) decreased, and the A5SS (20%), A3SS (26%) and ES (23%) increased in the sample of UF compared with that of YP (Fig. 1c). Then, the percentage of IR (34%) and A5SS (22%) were slightly elevated, accompanied with the decreased ES (18%), and A3SS (25%) in sample of F compared with that of UF (Fig. 1d). It’s noteworthy that MEE alternative splicing types comprised 1% among all the three samples, indicating that MEE alternative splicing type was stable during floret development (Fig. 1b–d). To verify the reality of the splicing events, 14 AS isoforms were selected randomly to check each isoform in RT-PCR. Amongst 14 AS isoforms, 11 were detectable at the mRNA level either with changed transcript levels or new isoforms (Fig. 2, Additional file 1: Fig. S3).

### Analysis of Differential Expressed AS (DAS) Events

**Fig. 3:**
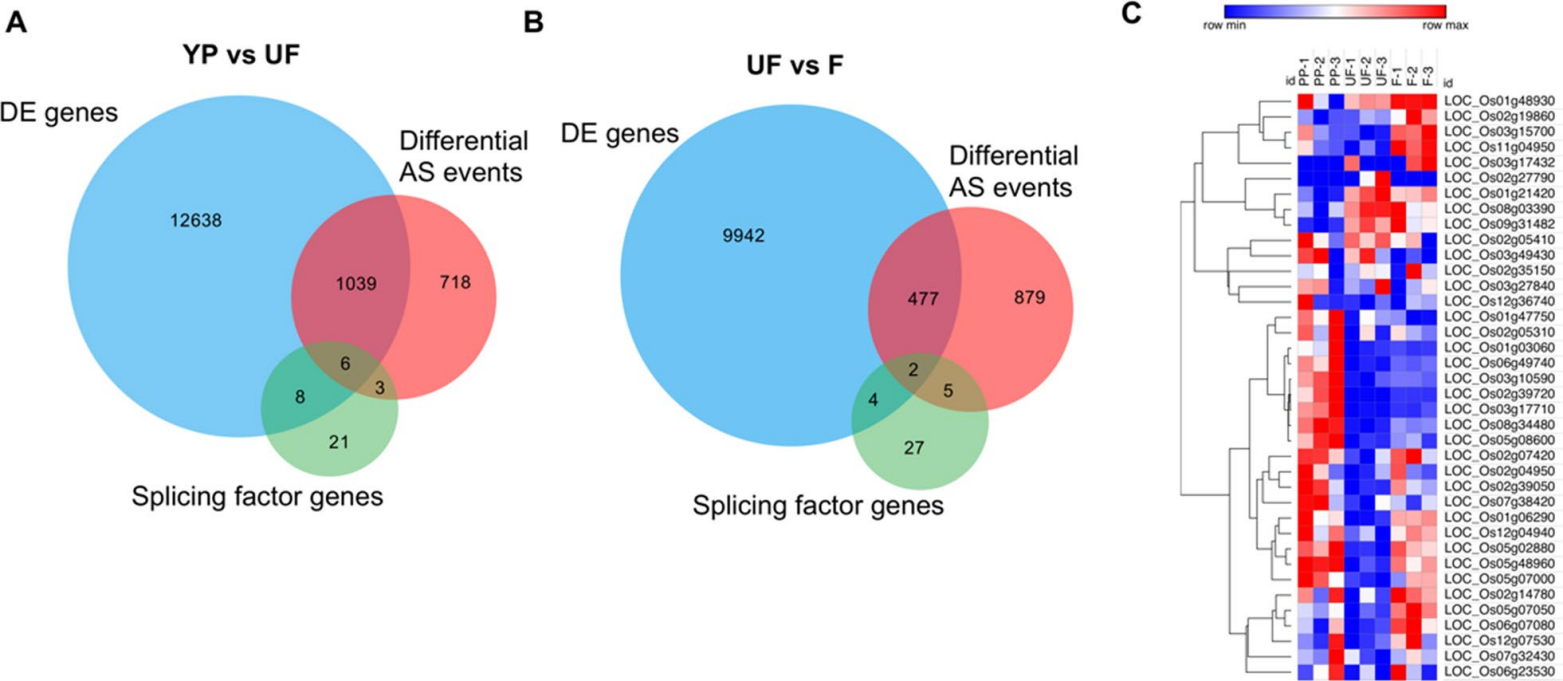
Comparison between differentially expressed (DE) genes and differentially AS genes. **a** Venn diagram showed number of DE genes associated with differential AS genes and splicing factors in the comparison of YP versus UF. Differentially expressed and differentially expressed AS events derived from the splicing factor genes were identified. **b** Venn diagram showed number of DE genes associated with DAS genes and splicing factors in the comparison of UF versus F. Differentially expressed and differentially expressed AS events associated with the splicing factor genes were identified. **c** Expression pattern of splicing factors showed in heat map. Gene ID of spicing factors were listed in the heat map. The blue color represents low expression levels, and the red color represents the high expression levels

To examine the function of floret development related alternative splicing genes in detail, Kyoto Encyclopedia of Genes and Genomes (KEGG) pathway analysis was conducted in each of the three samples. Firstly, the alternative splicing transcripts in YP were significantly enriched in “spliceosome”, “metabolic pathways” and “ribosome”, indicating that genes responsible for alternative splicing events were also alternatively spliced in YP (Additional file 1: Fig. S4A). Then, the most three abundant KEGG pathways in UF, were “metabolic pathways”, “spliceosome”, and “ribosome”, which was the same as that in YP (Additional file 1: Fig. S4B). To be noted that the pathway of “plant hormone signal transduction” was significantly enriched before flowering, suggesting the hormone related genes were alternatively spliced before double fertilization (Additional file 1: Fig. S4B). For those spliced events occurred after double fertilization in F, the alternatively spliced genes were significantly enriched in the pathway of “metabolic pathways”, “spliceosome”, and “biosynthesis of secondary metabolites” (Additional file 1: Fig. S4C). In addition, nitrogen metabolism related genes were also alternatively spliced at this time point, revealing that the alternative splicing events of nitrogen metabolism related genes played essential roles after double fertilization (Additional file 1: Fig. S4C). A total of 13,691 differentially expressed (DE) genes were identified in the comparison of UF_vs_YP. And 10,425 DE genes was identified in the comparison of F_vs_UF. DE genes might also alternatively spliced. Thereby, we compared the DE genes with differentially alternative splicing genes to identify differential expressed AS (DAS) events. Subsequently, the DAS events were identified in the comparison of UF_VS_YP and F_VS_UF, respectively (Fig. 3a, b). In total, 1045 AS transcripts were differentially expressed in the comparison of UF_vs_YP, and those DAS genes were enriched in the GO terms of “catalytic activity”, “transporter activity”, “binding”, and “nucleic acid binding transcription factor activity” (Fig. 3a, Additional file 1: Fig. S5). In contrast, much less AS transcripts were differentially expressed in the comparisons of F_vs_UF (Fig. 3b). The GO analysis of those DAS genes showed that terms of “catalytic activity”, “transporter activity”, and “binding”, excluding “nucleic acid binding transcription factor activity” were significantly enriched (Fig. 3b, Additional file 1: Fig. S5). In addition, one AP2 domain containing protein encoded by LOC_Os02g51300 was the differentially expressed AS among the three samples, which was validated by RT-PCR (Fig. 2). LOC_Os02g10920 encoding a zinc finger family protein displayed two transcripts in UF and F, while only one transcript in YP. Moreover, the transcript levels in UF and F increased highly in UF and F. We thereby proposed that alternatively spliced transcript of LOC_Os02g10920 played different roles in regulating floret development from the young panicle to post-fertilization process.

### Alternative Spliced Splicing Factors are Essential for AS

Splicing factors play key roles in guiding tissue-specific development processes (Thatcher et al. 2016). Alternative splicing difference among different tissues is thought to be the result of differentially expressed splicing factors and is likely to be influenced by tissue-specific methylation patterns (Regulski et al. 2013). Hence, the expression level of splicing factors was essential in modulating the alternative splicing events. In this study, a total of 38 splicing factor related genes were detected in the three samples according to the protein annotation (Fig. 3c), in which 14 DEs were either up or down regulated in the comparison of UF_vs_YP, and 6 DEs were identified in the comparison of F_vs_UF (Fig. 3a, b). Nevertheless, 9 splicing factors were alternatively spliced in UF compared with YP, in which 6 splicing factors were the DAS genes (Fig. 3a). Besides, 7 splicing factors were alternatively spliced, whereas only 2 DAS genes were identified in the comparison of F_vs_UF (Fig. 3b). Expression pattern of all the splicing factors expressed in the three samples were presented in the heat map (Fig. 3c). Previous evidence showed that splicing factors were alternative spliced frequently, leading to an increased or decreased number of alternative splicing events in their targeted genes (Zhang and Mount 2009; Li et al. 2020). Therefore, it’s reasonable that differentially expressed alternative splicing factors may result in the differentially expressed AS genes during floret development. In addition, one SR repressor protein encoded by LOC_Os12g38430 was alternatively spliced in the three developmental stages, which might be significant for the developmental stage transition.

### Effect of Alternative Spliced Transcripts on Rice miRNA Targets

**Fig. 4:**
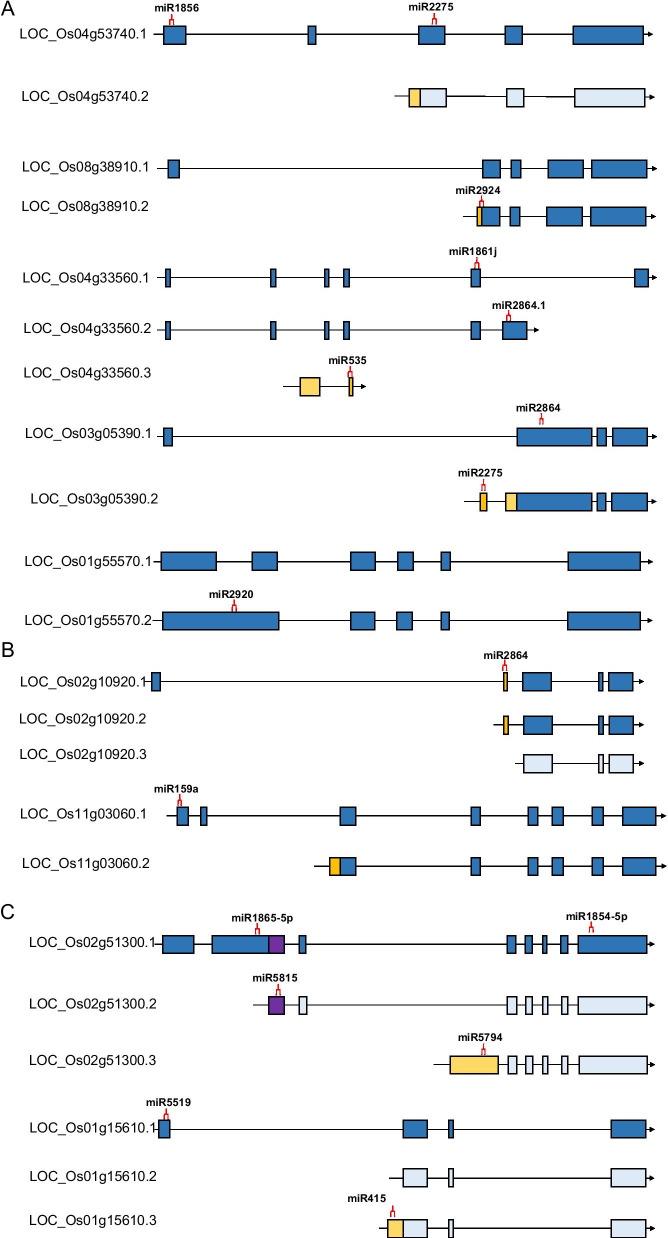
Gain and Loss of miRNA Target Sites in the alternative spliced transcripts. **a** miRNAs loss or gain targets site caused by the intron retention (IR) alternative splicing events. **b** miRNAs could loss or gain targets site caused by the exon skipping (ES) alternative splicing events. **c** miRNAs could loss or gain targets site because of the either IR or ES alternative splicing events. The miRNA target sites were indicated in the red symbols

miRNA-targets interaction usually repressed the transcript levels of target genes through guiding cleavage of target miRNAs by base-pairing. To assess how miRNAs interact with the alternative splicing, we performed the miRNA-targets prediction against all the transcripts identified in this study by using previous described methods with modification (Dai and Zhao 2011). In total, 1648 genes alternatively spliced were the predicted targets of known rice miRNAs (Additional file 2: Table S3). Many of these predicted targets were the IR type, displaying the lost or gained target sites. Then, we checked the miRNA binding sites of some AS genes, which verified by RT-PCR (Fig. 2). Results showed that ten out of eleven AS genes were targeted by different miRNAs through gain or loss targeting sites. For example, the second isoform of LOC_Os04g53740 lost the miR1856 targeting site (Fig. 4a). Transcript of LOC_Os08g38910.2 was targeted by miR2924 in the IR type (Fig. 4a). And the transcripts of LOC_Os04g33560.2 and LOC_Os04g33560.3 were targeted by miR2864.1 and miR535, respectively (Fig. 4a). Gene of LOC_Os03g05390 also gained the miR2275 and miR2864 targeting sites because of the IR types transcription (Fig. 4a). Interestingly, miR2775 was proposed to trigger phasiRNA production in premeiotic and meiotic anthers, which might responsible for the male fertility (Sun et al. 2018; Li et al. 2019; Xia et al. 2019), suggesting its role in targeting LOC_Os03g05390 to mediate anther development. The second isoform of LOC_Os01g55570 gained miR2920 targeting sites (Fig. 4a). ES transcripts could lose the targeting sites because of the alternative splicing events, for example, genes of LOC_Os02g10920 and LOC_Os11g03060 lost the targeting sites of miR2864 and miR159a, respectively (Fig. 4b). ES and IR types existed together also caused the gain or loss of targeting sites (Fig. 4c). These results suggested that miRNA-targets interactions could be affected by alternative splicing events.

### Association Between lncRNA and AS Genes

**Fig. 5:**
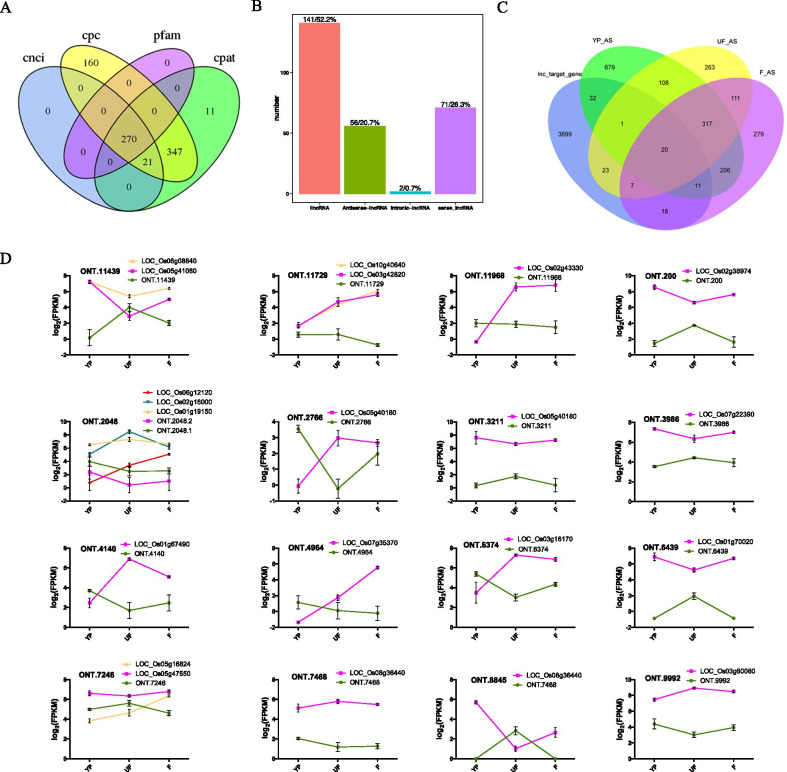
Association between lncRNA and alternative splicing (AS) genes. **a** Venn diagram showed the number of predicted lncRNAs using four methods. **b** Percentage of the four lncRNA types among the total predicted lncRNAs. **c** Comparison of the lncRNA predicted genes with AS corresponding genes in three developmental stages. **d** Examples of the expression pattern of lncRNA-AS interactions. Error bar represents mean ± SD with three biological replicates

Long noncoding RNAs (lncRNAs) participate in the regulations of transcription, splicing, and nuclear structure in plant (Chekanova 2015). In this study, we predicted new lncRNAs based on the novel transcripts identified in the dRNA-seq. In total, we identified 270 new lncRNAs based on four prediction methods, which contain anti-sense lncRNA (20.7%), intronic lncNRA (0.7%), lincRNA (52.2%) and sense lncRNA (26.3%) (Fig. 5a, b). Then we predicted its target genes according to the lncRNA-targets interaction mode, including the relative position of lncRNA and mRNA which differentially expressed per 100kbp on chromosomes, and complementary base pairing between lncRNA and mRNA. To examine how many genes were targeted by lncRNAs, which resulted in alternative splicing events, a comparison between lncRNAs targeted genes and alternative splicing genes was conducted (Fig. 5c). A total of 64, 51 and 56 lncRNAs targets were alternatively spliced in YP, UF, and F, respectively. Among all the lncRNAs, which targeted alternative splicing genes, lncRNAs of ONT.11439.1 repressed its potential targets of Histone H3 and RNA recognition motif containing protein in each of the three development stages (Fig. 5d). lncONT.200.2 and lncONT.3986.1 were predicted to interact with gene encoding 14-3-3 protein and gene encoding ankyrin repeat domain containing protein, resulting in alternative splicing of those genes respectively across the three stages (Fig. 5d). In addition, lncRNA could target different genes at different stages during floret development. lncONT.2048.1, targeted genes, which encoded CDA, MAPK, GSK3, and CLKC kinases in YP, and genes responsible for E1-BTB1—Bric-a-Brac, Tramtrack, and Broad Complex domain with E1 subfamily in UF. While BRASSINOSTEROID INSENSITIVE 1-associated receptor kinase 1 precursor gene was also targeted by lncONT.2048.1 in F.

### Analysis of Alternatively Spliced Transcription Factors (TFs)

**Fig. 6:**
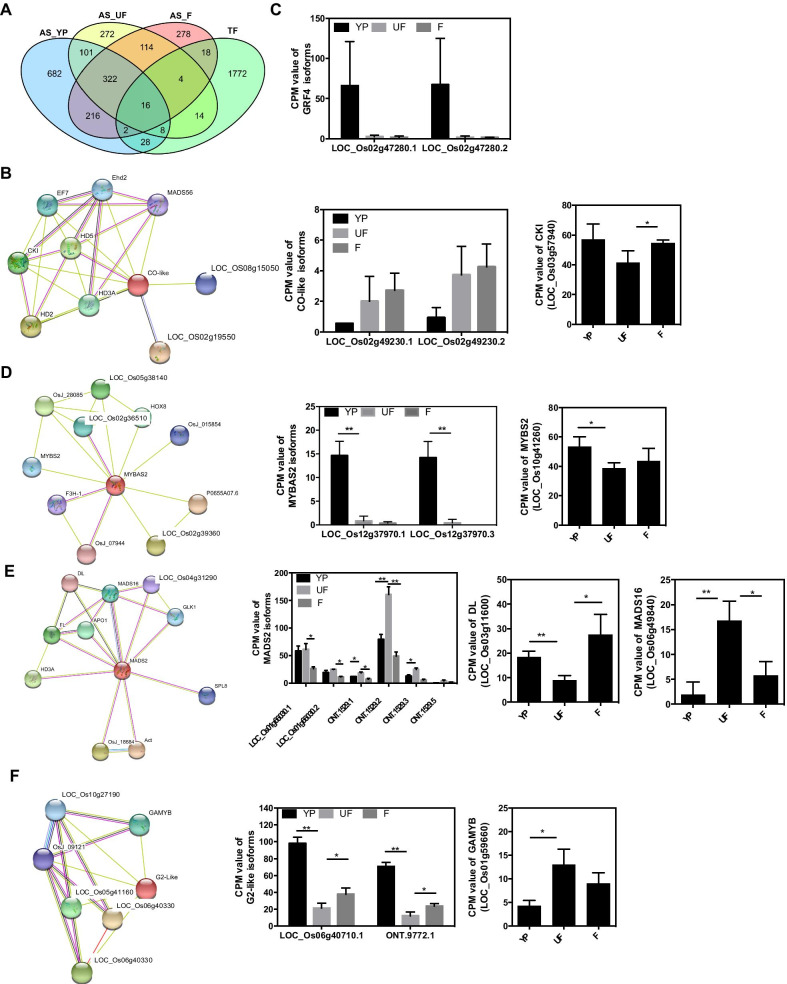
Analysis of alternative spliced transcription factors (TFs). **a** Venn diagram showed number of alternative spliced TFs in three developmental stages. **b** Co-like TF was predicted to interact with *CKI*, and expression levels of its two isoforms at three stages. **c** Expression levels of two *GRF4* isoforms. **d** MYBAS2 TF was interacted with *MYBS2* and expression levels of its two differentially expressed isoforms at three stages. **e** MADS2 was predicted to interact with *DL* and *MADS16*, and expression levels of all the six differentially expressed isoforms at three stages, among which ONT.1529.1, ONT.1529.2, ONT.1529.3, and ONT.1529.5. were the novel isoforms. Transcript levels of *DL* and *MADS16* were showed. **f** G2-like was predicted to interact with *GAMYB* and its expression levels at three stages. One novel isoform of ONT.9772.1was identified. The targeted gene expression level of *GAMYB* was showed. The prediction was conducted in the website of https://string-db.org/cgi/input.pl. Error bar represents mean ± SD with three biological replicates. Significance of *p < 0.05, **p < 0.01 were determined by Student’s t-test.  colored nodes: query proteins and first shell of interactors.  white nodes: second shell of interactors.  empty nodes: proteins of unknown 3D structure.  filled nodes: some 3D structure is known or predicted.  known interactions from curated databases.  known interactions from experimentally determined.  predicted interactions from gene neighborhood.  predicted interactions from gene fusions.  predicted interactions from gene co-occurrence.  others from textmining.  others from co-expression.  others from protein homology

**Fig. 7:**
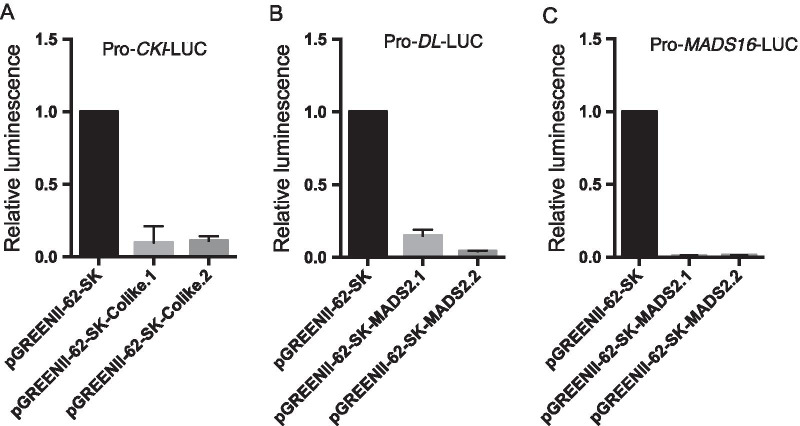
Validation the transcriptional inhibition role of alternative spliced transcription factors. **a** Coexpression of Colike and Colike.2 with LUC driven by the *CKI* promoter in protoplasts. Empty vector of pGREENII 0800-LUC mixed with the relative isoform was used as negative control. **b** Coexpression of MADS2 and MADS2.2 with LUC driven by the *DL* promoter in protoplasts. Empty vector of pGREENII 0800-LUC mixed with the relative isoform was used as negative control. **c** Coexpression of MADS2 and MADS2.2 with LUC driven by the *MADS16* promoter in protoplasts. Empty vector of pGREENII-62-SK mixed with the relative promoter of the targets were used as control. Bar graphs show means with two biological replications. Error bars show ± SD. The relative luminescence of control was set as 1.0

TFs are essential for plant development process, and the AS events associated with TFs are potentially important in regulating gene expression. In this study, we conducted the comparison analysis between AS genes and TFs, to detect alternatively spliced TFs during development (Fig. 6a). A total of 16 TFs were alternatively spliced among the three stages, which belonged to the family of ERF, WRKY, C3H, NAC, bZIP, Co-like, and etc. Amongst the 16 alternatively spliced TFs, Co-like encoded by LOC_Os02g49230, was predicted to interact with Casein kinase 1-like protein HD1 (CKI), which involved in development of male floral organs and grains, and flowering time under long day (Fig. 6b). Moreover, the transcript level of Co-like was increased during floret development, while expression level of CKI was opposite to that of Co-like, indicating that Co-like inhibited the expression of CKI (Fig. 6b). To validate whether Co-like co-expressed with CKI, we performed transient luciferase assay in the protoplasts. Results showed that both the two isoforms of Co-like, Colike.1 and Colike.2 inhibited the expression of the CKI (Fig. 7a, Additional file 1: Fig. S6A), which were consistent with its opposite expression pattern. Depending on Venn diagram, a total of 28 alternatively spliced TFs were specially identified in YP, in which 22 alternatively spliced TFs were either up or down regulated (Fig. 6a). Isoforms of GRF4.1 and GRF4.2 were significantly expressed in YP, suggesting its special role in the young panicle development (Fig. 6c). Previous evidence showed that GRF4 controls grain size and yield in rice (Duan et al. 2015). Therefore, the dramatically expressed isoforms of GRF4 could be important for the young panicle development. Isoforms of MYBAS2.1 and MYBAS2.3 were also significantly detected in the YP samples (Fig. 6d). And it was proposed to interact with *MYBS2* (LOC_Os10g41260), which was down regulated in YP, suggesting the negative correlation between *MYBS2* and MYBAS2 (Fig. 6d). TF of MADS2 encoded by LOC_Os01g66030 was predicted to interact with *MADS16* which regulated carpel specification in flower development, and *DL* which was required for normal development of lodicules and stamens (whorls 2 and 3) (Prasad and Vijayraghavan 2003) (Fig. 6e). A total of six isoforms of MADS2 were identified, among which ONT.1529.1, ONT.1529.2, ONT.1529.3, and ONT.1529.5. were its novel isoforms (Fig. 6e). Transcript levels of all the differentially expressed isoforms of *MADS2* showed similar expression pattern that *MADS2* was positively correlated with that of *MADS16*, whereas displayed a negative relationship with *DL* (Fig. 6e). Then, we performed the luciferase assay to validate whether MADS2 transactivate or inhibit expression of *DL* and *MADS16*. Results showed that *MADS2.1* and *MADS2.2* inhibited the *DL*, which was consistent with the negative correlations between transcript levels (Fig. 7b, Additional file 1: Fig. S6B). In contrast, *MADS16* was also inhibited by the two isoforms of *MADS*, which was not consistent with the positive correlations between expression levels (Fig. 7c, Additional file 1: Fig. S6C). In addition, G2-like transcription factor was predicted to interact with *GAMYB*, which showed opposite expression pattern between *G2-like* and *GAMYB* (Fig. 6f). Furthermore, the novel isoform of ONT.9772.1 derived from *G2-like* showed the similar expression pattern to the known isoforms of *G2-like.1* (Fig. 6f)*.* There were 14 TFs and 19 TFs uniquely alternatively spliced in UF and F, which might be also essential for the corresponding development (Fig. 6a).

## Discussion

Floral organogenesis, sex cell development before fertilization and post-fertilization affect crop yield, thereby, a better understanding of the regulatory mechanisms in reproductive development is essential for improvements in agricultural practice (Sunkar and Jagadeeswaran 2008; Huijser and Schmid 2011). However, gene regulation at the post-transcriptional level is less understood in terms of rice floret development. AS regulates plant biological processes from plant development to stress responses, while developmental processes such as the flowering time and circadian clock are strictly affected by AS (James et al. 2012; Rosloski et al. 2013; Feng et al. 2015; Zhu et al. 2017). Investigations within the AS analysis could lead to the much more accurate gene functions in plant developmental process. Therefore, AS might be significant for the rice yield production through the regulation of male and female organ development, and the process of double fertilization. The differentially expressed AS genes, would provide deeper insight into the functional importance of AS during plant biological development process. Therefore, increasing our knowledge of gene expression pattern and regulatory network in terms of AS, and miRNA from young panicles to flowered florets in rice help to lay the foundation of improving crop yield. In this study, we carried out the full-length mRNA sequencing, and degradome sequencing, to elucidate AS events and its potential regulators involved in the floret development process. The DAS derived from differentially expressed genes in this study indicated that the AS might participate essential roles in the floret development before flowering and after flowering.

A comparison of alternative splicing types detected in the three stages revealed dynamic alternative splicing during florets development process (Fig. 1b–d). Previous studies in Arabidopsis and cassava uncovered that IR was the most prevalent AS types, which were consistent with our results (Filichkin et al. 2010; Li et al. 2020). The percentage of IR in YP was the largest, and decreased in UF and F in rice (Fig. 1b–d). DE genes during florets development were significantly enriched in spliceosome, plant hormone signal transduction and metabolic pathway. Interestingly, the AS events occurred in the florets at three stages were also enriched in spliceosome, plant hormone signal transduction and metabolic pathway, indicating that splicing events could alter the expression pattern of the corresponding genes involved in floret development. The stability of mature RNA usually decreased by alternative splicing, and the alternative splicing could also trigger nonsense-mediated decay (NMD), which was a process that influences the steady state levels of specific transcripts by targeting them for decay (Mühlemann 2008). AS coupled NMD was a major determinant in shaping the Arabidopsis transcriptome (Drechsel et al. 2013). DE genes in the comparison of UF_vs_YP enriched in the mRNA surveillance pathway, indicating the degradation of alternatively spliced transcripts during mRNA maturing (Kalyna et al. 2012). Thus, we performed the degradome-seq in YP and found a total of 751 degraded genes. The comparison of the degradome data and DAS genes revealed 93 degraded genes, accounting for ~ 6.8% of the total DAS genes. The low percentage of the degraded genes suggested a productive transcripts generation in YP, which potentially leads to high efficiency of protein translation (Additional file 1: Fig. S7).

lncRNA is able to establish and maintain cell specific alternative splicing via modulation of chromatin signatures (Gonzalez et al. 2015). Arabidopsis lncRNA modulates alternative splicing regulators, and hijacks them to change alternative splicing patterns to produce AS events (Bardou et al. 2014). In our work, a total of 270 new lncRNAs were identified in developing florets of rice. lncRNAs targeted genes could be overlapped with the AS at the three developmental stages. Notably, lncONT.200.2 and lncONT.3986.1were predicted to interact with gene encoding 14-3-3 protein, while 14-3-3 protein was reported to interact with REPRESSION OF SHOOT GROWTH (RSG) to regulate the spikelets size (Jang et al. 2017). The correlation between the lncRNA and AS suggested a potential role of lncRNA in regulating alternative splicing.

Floret development is a complex process that transcription factors are essential for gene transcription in this process, while the alternative spliced TFs possibly affect many genes. TFs areimportant in controlling grain filling in crops. For example, transcription factor of NAC transactivates expression of starch synthesis gene in rice (Wang et al. 2019a). Rice basic leucine zipper factor and rice prolamin box binding factor activates the transcription of seed storage protein genes, which subsequently cause starch accumulation in seeds (Liu et al. 2018a). In addition, OsSPL16 (GW8) binds directly to the GW7 promoter and represses its expression, thus affects grain yield (Wang et al. 2015). In our study, more than half of the commonly alternative splicing TFs were differentially expressed. Among those TFs, Co-like interacted with CKI to regulate development of male floral organs and grains, indicating its crucial roles across the three developmental stages (Figs. 6b, 7a). Additionally, some unique differential expressed and alternatively spliced TFs in YP, for example, GRF4, MYBAS2 and MADS2 were also predicted to interact with genes involved in young panicle development and grain size (Prasad and Vijayraghavan 2003; Li et al. 2016). Thus, these mentioned TFs might be pivotal in young panicle development. Therefore, further molecular and genetic studies are necessary to validate roles of the candidate TFs in young panicle development.

In conclusion, we investigated the complex networks of AS associated with degradome-seq, miRNAs, and lncRNA, which provide insights into comprehensive understanding of floret development in the posttranscriptional regulation level. The discovery of the alternative splicing events, including the miRNA targets and TFs, increase our knowledge of alternative splicing mechanisms during rice florets development.

## Materials and Methods

### Plant Materials and Sampling

The rice cultivar 4266 was provided by the Rice Research Institute, Guangdong Academy of Agricultural Sciences, and planted in the greenhouse of The Chinese University of Hong Kong, Hong Kong, China, during the rice growing season (from March to August 2019). The young panicles (YP, 1 mm–1 cm), unfertilized florets (UF) and fertilized florets (F) were sampled and were immediately frozen in liquid nitrogen and stored at − 80 °C for further experiments.

### RNA Extraction and Library Construction for Direct RNA Sequencing (dRNA-seq) Using Nanopore

The total RNA from young panicles (YP), unfertilized florets (UF) and fertilized florets (F) were extracted using the Trizol Reagent (Life technology). The library preparation was followed with the standard protocol of Oxford Nanopore Technologies (ONT) and the sequencing was carried out by sequencing company (Biomerker) (Deamer et al. 2016). More than 2.5 GB clean data were obtained in each sample. The raw data was trimmed to remove adaptor sequences and filtered, the length of these clean tags which was longer than 500 bp were mapped to japonica (MSU7) by minimap2 (Li 2018). Software of pinfish was deployed to obtain consensus isoforms and then the minimap2 was used to remove the redundant isoforms. After that the redundancy removed consensus isoforms can be used for alternative splicing analysis. The expression level was calculated by using counts per million (CPM) accordingly (Cui et al. 2020). Astalavista was used to determine alternative splicing types in each sample (Foissac and Sammeth 2007). DESeq was used to determine differentially expressed (DE) genes and differentially expressed alternative splicing (DAS) events with the cutoff of log_2_FC > 2 and q-value (false discovery rate, FDR < 5%).

### Degradome Processing

Poly(A) RNA was purified from total RNA (20ug) of YP using poly-T oligo-attached magnetic beads using two rounds of purification. Then transcription was reversed to make the first strand of cDNA with a 3′-adapter random prime (150 ng of poly(A) + RNA was used), and 5′ adaptor ligation to those RNAs only containing 5′-monophosphates. Then the cDNA was amplified by PCR. The average insert size for the final cDNA library was 200–400 base pair (bp). Libraries were sequenced using the 5′ adapter only, resulting in the sequencing of the first 36 nucleotides of the inserts that represented the 5′ ends of the original RNAs. At last, we performed the 50 bp single-end sequencing on an Illumina Hiseq 2500 (LC Bio, China) following the vendor's recommended protocol. The extracted sequencing reads were then used to identify potentially cleaved targets by the CleaveLand pipeline. All resulting reads (t-signature) were reversely complemented and aligned to the miRNA identified in our study. The degradome sequence position, which coincident with the tenth or eleventh nucleotide of miRNA were retained and scored.

### Long Non Coding RNA (lncRNA) Analysis and the Prediction of Its Targets

New lncRNAs among all the dRNA-seq reads was determined based on its potential in protein coding by using four methods CPC (Coding Potential Calculator) (Kang et al. 2017)(Kang et al. 2017), CNCI (Coding-Non-Coding Index) (Li et al. 2014), CPAT (Coding Potential Assessment Tool) (Wang et al. 2013) and pfam (Finn et al. 2016). lncRNAs were then obtained based on the intersection of all the identified lncRNAs determined by the above four methods. Targets of lncRNAs were predicted with two methods: (1) prediction of LncRNA target genes based on location relationship and (2) target genes prediction based on complementary sequence using LncTar (Li et al. 2015).

### Identification of Rice miRNA Targets

The in-silico interactions between candidate targets and published miRNAs in rice were predicted by the scoring schema V2 from psRNATarget web service at [http://plantgrn.noble.org/psRNATarget] with default parameters, except that we set the maximum cutoff ‘Expectation’ as 10 to access more potential miRNA-target interactions based on the scoring rule (Dai and Zhao 2011).

### Reverse Transcription (RT)-PCR Validation of Alternative Spliced Transcripts

Total RNA was reversely transcribed using Superscript First-strand synthesis system (Invitrogen), following the manufacturer’s protocol. RT-PCR was then conduted with twenty-five rounds of PCR amplification using Taq polymerase, while *OsActin2* was used as control. After the amplification, gel visualization was conducted to verify its expression pattern. The specific primers used here are listed in Additional file 2: Table S4.

### Analysis of Luciferase In Vivo

The sequence of the native promoters of *CKI, DL,* and *MADS16* (*Pro-CKI, Pro-DL, Pro-MADS16*), were amplified from 4266 genomic DNA, respectively. The amplified promoters were cloned into the *pGREENII-0080-luc* vector by a one-step cloning kit (Vazyme, Nanjing, China) using enzyme sites of KPNI and BamHI, to form the reporter constructs. Then, the CDS region of the *Colike.1*, *Colike.2*, *MADS2*,0.1 and *MADS2.2* were amplified and cloned into the *pGREENII-62-SK* vector by a one-step cloning kit (Vazyme, Nanjing, China) using BamHI and KPNI, to form the effector constructs. Then, the above constructed vectors were mixed well for the transient expression assay in the rice protoplasts. This transient expression assay was performed as described previously (Wang et al. 2019a, b; Wang et al. 2020). The primers used were listed in Additional file 2: Table S5.

### Data Analysis

Statistical analysis was performed in the Graph prism 6 software.

## Supplementary Information

**Additional file 1: Figure S1**. Quality of the clean reads. **Figure S2**. Mapping rate of identified new genes and transcripts and its Gene Ontology (GO) enrichment.**Figure S3**. Transcript levels of validated AS isoforms relative to Fig. 2. **Figure S4**. Kyoto Encyclopedia of Genes and Genomes (KEGG) analysis of alternative spliced (AS) events in three stages. **Figure S5**. Gene Ontology (GO) analysis of differentially alternative spliced (DAS) genes in the comparisons of YP vs UF and UF vs F. **Figure S6**. Validation the transcriptional inhibition role of alternative spliced transcription factors using another control. **Figure S7**. Comparisons of degradome-seq data and differential AS transcripts of young panicle (YP)

**Additional file 2: Table S1**. Summary of the clean data. **Table S2**. Summary of the identified transcripts and genes, and its position. **Table S3**. A total of 1648 genes alternatively spliced were targeted by miRNA in rice. **Table S4**. Primers used for validating alternative splicing events by RT-PCR. **Table S5**. Primers used in the luciferase assay in vivo

## Data Availability

Sequence data from this article can be found in the GenBank/EMBL data libraries under accession numbers PRJNA644762.
