# Supplementary material for: Global Survey of Alternative Splicing in Rice by Direct RNA Sequencing During Reproductive Development: Landscape and Genetic Regulation
Source: Rice (N Y). 2021 Aug 12;14:75. doi: 10.1186/s12284-021-00516-6 (PMC8360254; doi:10.1186/s12284-021-00516-6)
Supplement: Supplementary file 1 — Additional file 1: Figure S1. Quality of the clean reads. Figure S2. Mapping rate of identified new genes and transcripts and its Gene Ontology (GO) enrichment.Figure S3. Transcript levels of validated AS isoforms relative to Fig. 2. Figure S4. Kyoto Encyclopedia of Genes and Genomes (KEGG) analysis of alternative spliced (AS) events in three stages. Figure S5. Gene Ontology (GO) analysis of differentially alternative spliced (DAS) genes in the comparisons of YP vs UF and UF vs F. Figure S6. Validation the transcriptional inhibition role of alternative spliced transcription factors using another control. Figure S7. Comparisons of degradome-seq data and differential AS transcripts of young panicle (YP) [file 12284_2021_516_MOESM1_ESM.docx]

**Supplemental Figure S1. Quality of the clean reads.**

A, Quality distribution of clean reads in different base positions. B, Sequencing reads length and average quality value in different reads length range.

**Supplemental Figure S2. Mapping rate of identified new genes and transcripts and its Gene Ontology (GO) enrichment.**

A, Mapping rate of novel genes to rice and other species. B, GO enrichment analysis of the novel genes in terms of cellular component, molecular function and biological process. C, Mapping rate of novel transcripts to rice and other species. D, GO enrichment analysis of the novel genes in terms of cellular component, molecular function and biological process.

**Supplemental Figure S3. Transcript levels of validated AS isoforms relative to Figure 2.**

All the graphs were plotted using the CPM values determined by dRNA-seq. The significant analysis was determined by Student’s t-test. * represents p<0.05; **represents p<0.01; ns stands for no significant.

**Supplemental Figure S4. Kyoto Encyclopedia of Genes and Genomes (KEGG) analysis of alternative spliced (AS) events in three stages.**

A, KEGG enrichment of young panicle (YP). B, KEGG enrichment of unfertilized florets (UF). C, KEGG enrichment of fertilized florets (F).

**Supplemental Figure S5. Gene Ontology (GO) analysis of differentially alternative spliced (DAS) genes in the comparisons of YP vs UF and UF vs F.**

A, GO terms analysis of up regulated DAS in UF compared with YP. B, GO terms analysis of down regulated DAS in UF compared with YP. C, GO terms analysis of up regulated DAS in F compared with UF. D, GO terms analysis of down regulated DAS in F compared with UF.


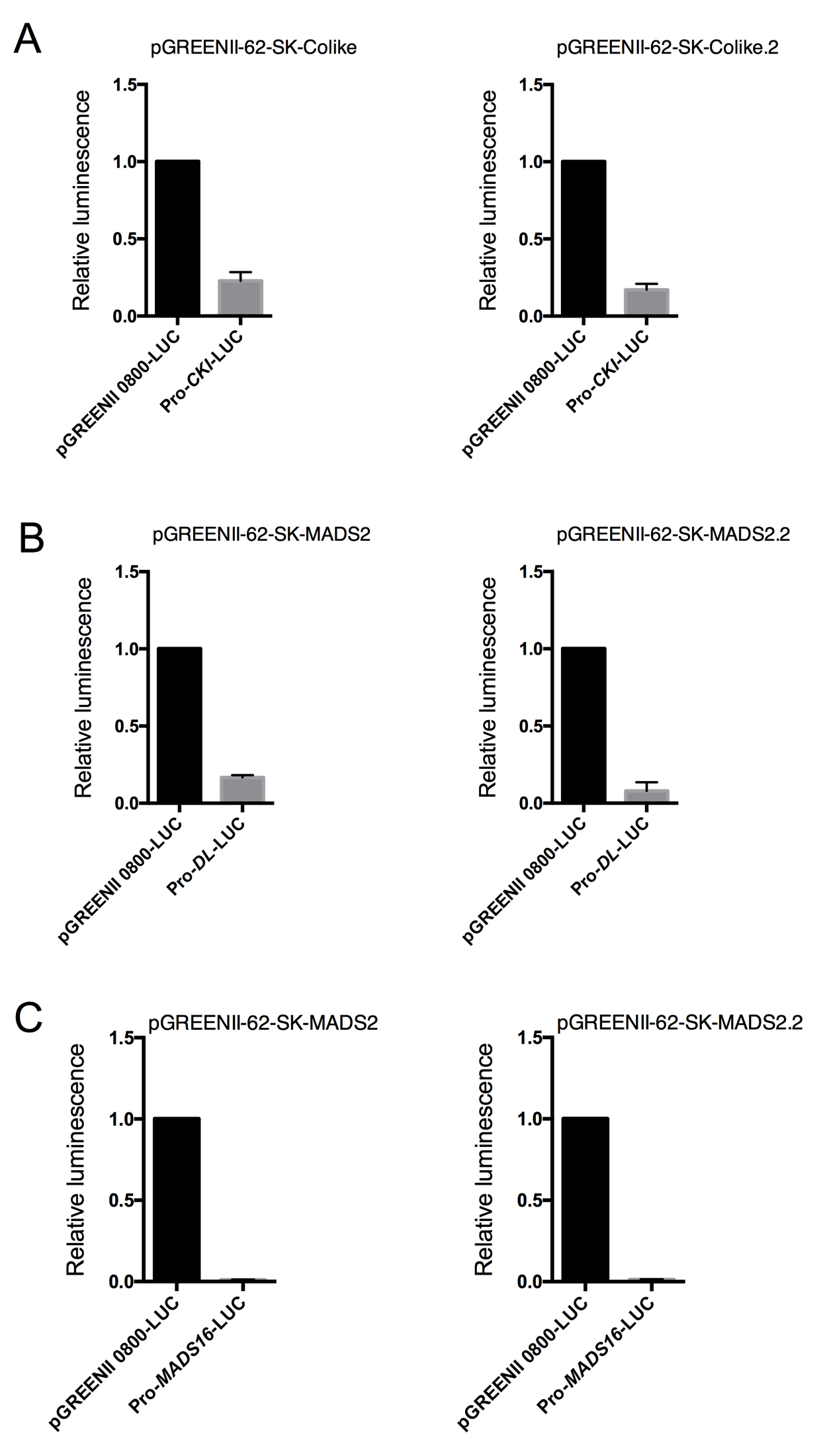


**Figure S6. Validation the transcriptional inhibition role of alternative spliced transcription factors using another control.**

(A) Coexpression of Colike and Colike.2 with LUC driven by the *CKI* promoter in protoplasts. Empty vector of pGREENII 0800-LUC mixed with the relative isoform was used as negative control. (B) Coexpression of MADS2 and MADS2.2 with LUC driven by the *DL* promoter in protoplasts. Empty vector of pGREENII 0800-LUC mixed with the relative isoform was used as negative control. (C) Coexpression of MADS2 and MADS2.2 with LUC driven by the *MADS16* promoter in protoplasts. Empty vector of pGREENII 0800-LUC mixed with the relative isoform was used as control. Bar graphs show means with two biological replications. Error bars show ± SD. The relative luminescence of control was set as 1.0.

**Supplemental Figure S7. Comparisons of degradome-seq data and differential AS transcripts of young panicle (YP).** Venn diagram showing DAS events in YP and corresponding genes detected by the degradome-seq. The overlapped 93 genes were the degraded genes with DAS events.
